# Sentinel-2 image transformation methods for mapping oil spill – A case study with Wakashio oil spill in the Indian Ocean, off Mauritius

**DOI:** 10.1016/j.mex.2021.101327

**Published:** 2021-03-27

**Authors:** Sankaran Rajendran, Ponnumony Vethamony, Fadhil N. Sadooni, Hamad Al-Saad Al-Kuwari, Jassim A. Al-Khayat, Himanshu Govil, Sobhi Nasir

**Affiliations:** a Environmental Science Center, Qatar University, P.O. Box: 2713, Doha, Qatar; b Department of Applied Geology, National Institute of Technology, Raipur, India; c UNESCO Chair for Ophiolite Studies, Sultan Qaboos University, Al-Khod, 123 Muscat, Oman

**Keywords:** Sentinel-2, Image processing, Indices, Decorrelation stretch, Oil spill thickness

## Abstract

Although several indices have been constructed and available at the Index database (IDB) for Sentinel-2 satellite to map and study several earth resources, no indices have been developed to map oil spill. We constructed band ratios (5 + 6)/7, (3 + 4)/2, (11+12)/8 and 3/2, (3 + 4)/2, (6 + 7)/5 using the high-resolution MSI (multi-spectral instrument) visible-near infrared-shortwave infrared spectral bands of Sentinel-2 by summing-up the bands representing the shoulders of absorption features as numerator and the band located nearest to the absorption feature as denominator to discriminate oil spill, and demonstrate the potential of this method to map the Wakashio oil spill which occurred in the Indian Ocean, off Mauritius. The resulted images discriminated the oil spill well. We also decorrelated the spectral bands 4, 3 and 2 by studying the spectral band absorptions and discriminated the spill as very thick, thick and thin. The results of decorrelation stretch method exhibited the distribution of types of oil spill in a different tone, distinctly. Both the image transformation methods (band ratios and decorrelation stretch methods) showed their capability to map oil spills, and these methods are recommended to use for similar spectral bands of other sensors to map oil spills.

• This study demonstrated the application of band ratios and decorrelation stretch methods to map oil spill.
• The methods discriminated the oil spill off Mauritius, and showed spill thicknesses from the Sentinel-2 data.
• The new methods are recommended to use for the spectral bands of other sensors to map oil spill.

## Graphical abstract

**Figure:**
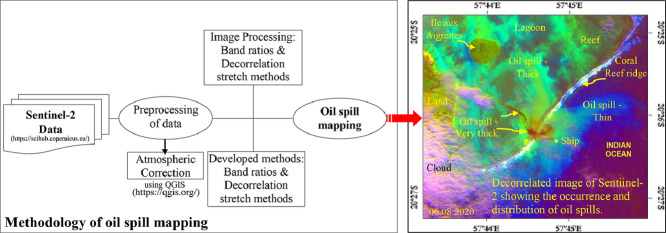

Specifications Table

**Table:** 

Subject Area:	Environmental Science
More specific subject area:	Oil spill - Marine Pollution
Method name:	Sentinel-2 image transformation methods for mapping oil spill
Name and reference of original method:	The capability of the methods, namely, band ratios and decorrelation stretch methods are demonstrated and proposed through this study by the authors.
Resource availability:	Data access: https://sentinel.esa.int/web/sentinel/sentinel-data-access Software used: ENVI 5.5 software https://www.harrisgeospatial.com

## Introduction

**Fig. 1:**
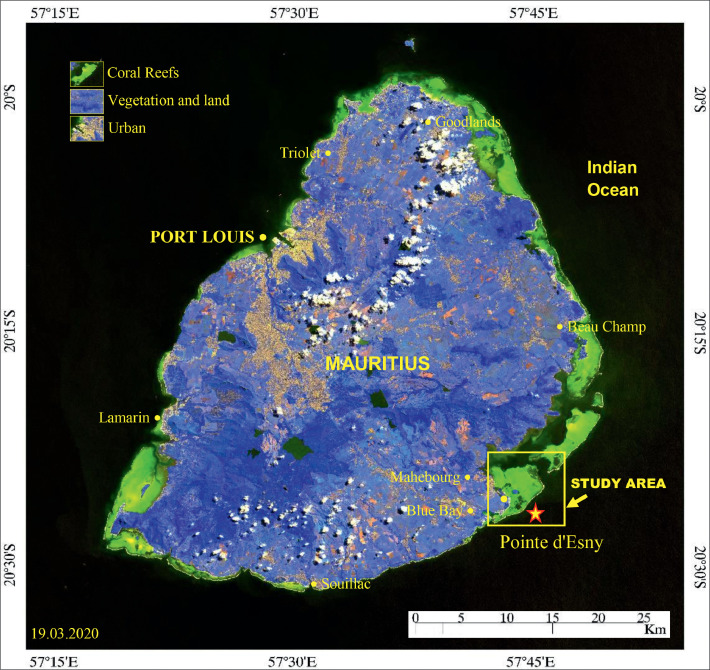
False-Color Composite (FCC) of MSI (R:8; G:4; B:3) of March 19, 2020, showing the study area (dashed rectangle) and the occurrence and distribution of major features such as urban (maroon), coral reefs (green), and land and vegetation (blue) in and around the island in the Indian Ocean. Star locates oil spill site. (For interpretation of the references to color in this figure legend, the reader is referred to the web version of this article.)

Oil spills mapping is essential to evaluate the potential spread of the spill from the source to the adjacent areas or endpoints and assess the impact of the area. The detection of oil spills utilizing the visible, shortwave to thermal infrared (optical) and microwave radar bands have reached remarkable advances. Studies demonstrated the capability of Synthetic Aperture Radar (SAR) bands to detect marine oil spills, although the images have difficulties in showing the oil spill thickness and distinguishing oil from water and issues of look-alikes [1,2,3]. To overcome this issue and validate the results of SAR [4,5], data of optical sensors have been used. The high spatial resolution (10, 20, and 60 m) multispectral bands of Sentinel-2 available since June 2015 have been used to map the oil spill very effectively [1,6]. The band combinations using MSI bands of Sentinel-2 for different applications are found in the Sentinel Application Platform (SNAP) program (https://custom-scripts.sentinel-hub.com/custom-scripts/sentinel-2/indexdb/). However, no band ratios or indices were developed for discriminating and mapping the thickness of oil spills and assessing the impacts. In this context, we have demonstrated the capability of image transformation methods, viz., band ratios and decorrelation stretch methods to map and assess the oil spills, for which, as a case study, the Wakashio oil spill that occurred in the Indian Ocean, off Mauritius on August 06, 2020, has been chosen (Fig. 1).

## Details of methods

### Image transformation methods

The image transformation methods applied to digital image processing of satellite data, change the original data to another data in space through a linear function. The methodology includes calculation of band ratios and then applying the decorrelation stretch, which allows to interpret the results better than the original data. In brief, the construction of *band ratios and indices* enhances the spectral differences between the bands and reduces the effects of topography. Division of one spectral band by another produces an image that provides relative band intensities; spectral differences between bands allow to interpret the image features to the best (Envi, 5.5). For example, Rajendran et al. [7,8] and Amer et al. [9] developed ASTER spectral band ratios to map geological formations. In this study, we constructed band ratios (5 + 6)/7, (3 + 4)/2, (11+12)/8 and 3/2, (3 + 4)/2, (6 + 7)/5 by summing up the bands representing the shoulders of absorption features in the numerator and the band located near the absorption feature in the denominator using the spectral bands of visible-near infrared-shortwave infrared regions of MSI to discriminate the oil spill from the spectral absorption characters of Sentinel-2 bands. The ratio (3 + 4)/2 was constructed using visible bands to increase the spectral response of oil spill over the image, the ratios (6 + 7)/5 and (5 + 6)/7 were developed using NIR bands to show the vegetation-related information and the ratio (11+12)/8 was constructed using SWIR bands to study the lithological information of the study area.

Moreover, the *decorrelation stretch* method was used to show the thickness of oil spills. The method removes the high correlation commonly found in multispectral datasets. It produces a more colorful composite image by transforming the highly correlated data-sets usually from three bands input. The transformed channels can themselves be contrast stretched, and primary colors can be arbitrarily assigned to display a color composite image [10,11]. The method has the potential to distinguish spectral reflectance of different features of the earth's surface and map rock types using enhanced Landsat Thematic Mapper data [12,13] and ASTER data [14,15]. In this study, we decorrelated the spectral bands 4, 3, and 2 of Sentinel-2 to demonstrate the potential of this method. Here, we selected bands 3 and 4 to differentiate the oil spill thickness and band 2 to discriminate the water from the oil spill.

### Data used

In this study, we used the MSI (multispectral instrument) spectral bands of Sentinel-2. Sentinel-2A (launched on June 23, 2015) and Sentinel-2B (launched on March 7, 2017) were launched into circular sun-synchronous orbits at an altitude of 786 km with 98.62° inclination. These satellites have equatorial crossing times of 10:30 a.m. with a phase delay of 180^°^ [16]. The data are acquired with a 20.6° field of view, providing approximately 290 km swath. The equatorial repeat cycle of each Sentinel-2 sensor is about 10 days, and five days when combined [17]. MSI data have high spatial (10, 20, and 60 m) and 12-bit radiometric resolutions. We used the spectral bands of visible-near infrared-shortwave infrared region. The data were georeferenced to the WGS-84 ellipsoid and Universal Transverse Mercator (UTM) Zone 40S projection. The pre-processing of data was carried out using QGIS (3.12.3-Bucuresti). Subsequently, the data were subset to the area of interest and images were processed using ENVI™ software (version 5.5, Harris Geospatial Solutions, Broomfield, CO, USA). In order to obtain the best results, the Sentinel-2 images were not processed for removing the clouds. In this study, the visual interpretation of images has been considered as one of the most effective methods to study the oil spill [18] and demonstrated the potential of image processing methods. All the Sentinel-2 data for the period of July 17-September 10, 2020, covering the spill event (before, during, and after) were downloaded freely from the Copernicus Open Access Hub (https://scihub.copernicus.eu/). The sensor characteristics of Sentinel- 2 are given in Table 1 and the data used to demonstrate the methods are given in Table 1 in Appendix-A.

**Table 1:** Sensor characters of Sentinel-2 according to the Copernicus derived user requirements (from van der Meer et al., 2014).

Bands	Wave length regions	Center Wavelength (nm)	Band width (nm)	Spatial Resolution (m)	Purpose
1	VNIR	443	20	60	Atm. correction (aerosol scattering)
2		490	65	10	Vegetation senescing, carotenoid, browning and soil background; atm. correction (aerosol scattering)
3		560	35	10	Green peak, sensitive to total chlorophyll in vegetation
4		665	30	10	Max. chlorophyll absorption
5		705	15	20	Red edge position; consolidation of atmospheric corrections/fluorescence baseline.
6		740	15	20	Red edge position; atmospheric correction; retrieval of aerosol load
7		783	20	20	LAI^a^; edge of the NIR plateau
8		842	115	10	LAI
8a		865	20	20	NIR plateau, sensitive to total chlorophyll, biomass, LAI and protein; water vapor absorption reference; retrieval of aerosol load and type
9		945	20	60	Atm. correction (water vapor absorption)
10	SWIR	1375	30	60	Atm. correction (detection of thin cirrus)
11		1610	90	20	Sensitive to lignin, starch and forest above ground biomass; snow/ice/cloud separation
12		2190	180	20	Assessment of Mediterranean vegetation conditions; distinction of clay soils for monitoring of soil erosion; distinction between live biomass, dead biomass and soil, e.g. for burn scars mapping

a LAI = Leaf Area Index.

### Spectral band absorptions of MSI

Previous works showed that the green and red bands of remotely sensed data are the optimal bands for detecting oil spills [19,20,21,22]. In this study, to develop band ratio and to discriminate the oil spill, we collected image spectra of MSI data and studied the spectral band absorptions of water and oil spill, since the library and field spectra were not acquired under the same conditions of the satellite images, and the image spectra are directly associated with the surface features that detectable on the image. The spectra of oil and seawater [23] and the spectra of MSI image acquired on August 6, 2020 for the locations offshore, lagoon, and oil spills are given in Fig. 1 in Appendix-A. The image spectra were collected by removing the bands 1, 9 and 10 that represent aerosol, water vapor and cirrus cloud. The study of the reflectance between oil and seawater showed the presence of high reflectance in oil when compared with the reflectance of seawater in the wavelength between 390 nm and 1000 nm (Fig. 1(a) in Appendix-A). The spectra of oil and seawater exhibited strong absorptions near 450 nm, weak absorptions around 600 nm and flat in the NIR region. Further, the study of spectra of water (Fig. 1(c) in Appendix-A) showed the occurrence of strong absorptions in the MSI spectral bands 4 (665 nm, red vertical line), 6 (740 nm, red dashed vertical line), 8a (865 nm, blue vertical line) and 11 (1610 nm, blue dashed vertical line), and exhibited variations in their reflectance (see the reflectance values). The spectra of offshore showed poor reflection due to strong absorption of light in the relatively deep water and spectra of shallow water of the lagoon showed high reflection due to less absorption of light in the shallow depths. Besides, spectra of lagoon having relatively larger depths showed high reflection when compared with the spectra of offshore, which may be due to the presence of coral reefs in the area.

The spectra collected over the oil spill areas showed strong absorptions in the spectral bands 3 (560 nm, red vertical line), 4 (665 nm, red dashed vertical line), 6 (740 nm, red dashed and pointed vertical line), 8a (865 nm, blue vertical line) and 11 (1610 nm, blue dashed vertical line) in the visible, NIR and SWIR regions (Fig. 1(d) in Appendix -A). The spectra of very thick oil spill showed absorptions in the bands 3, 4, 6, 8a, and 11, and the absorptions were strong in the bands 3, 4, and 6 and relatively poor in the bands 8a and 11 when compared with the thick and thin oil spills. The absorptions in bands 3 and 4 are due to the presence of oil on the water and the absorptions in bands 8a and 11 may be due to the influence of water vapor or water below the oil. The spectra of thick oil spills showed absorptions in bands 3, 4, 6, 8a, and 11 similar to the absorptions of the very thick oil spill. The spectra of the thin oil spills also exhibited similar absorptions in the bands but, showed strong absorptions in bands 6, 8a, and 11 when compared with the very thick and thick oil spills. The absorption in band 6 may be due to the presence of aerosol over the water. The spectra of very thick oil spills exhibited poor reflection when compared with the other types of oil spills; the thin oil spills showed high reflectance. This study suggests that the spectral bands 3 and 4 of MSI are best to use for image processing and discriminate oil spills [19,[21], [22], [23], [24], [25], [26], [27], [28]]. The spectral bands acquired in these wavelengths by other satellites can also discriminate oil spills.

### Sentinel-2 band ratios and discrimination of oil spill

**Fig. 2:**
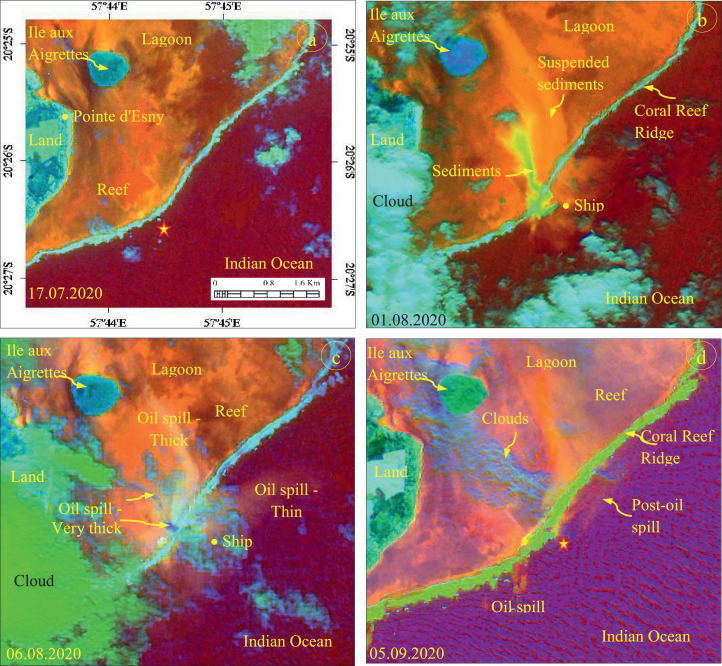
Band ratio R: (5 + 6)/7; G: (3 + 4)/2; B: (11+12)/8 images showing the occurrence and distribution of oil spill in the incident site and from the site to lagoon (Stars locate the incident site).

**Fig. 3:**
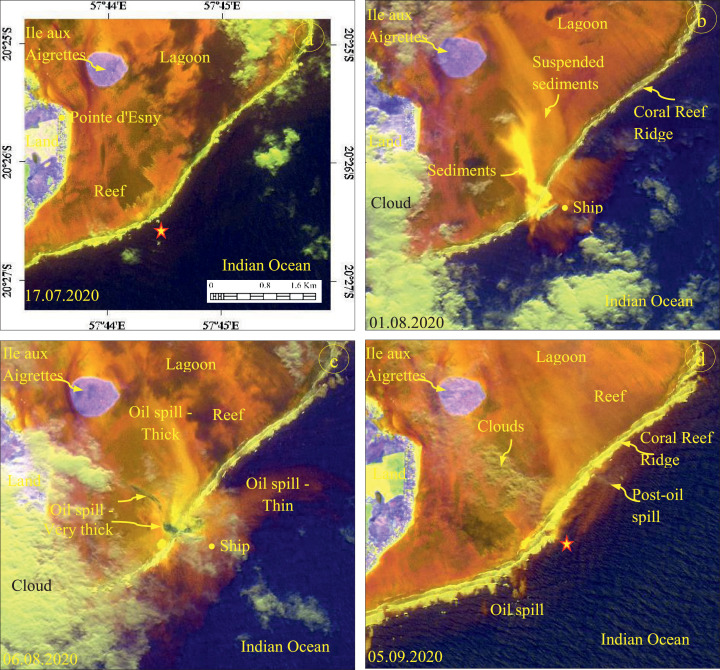
Band ratio R: 3/2; G: (3 + 4)/2; B: (6 + 7)/5 images showing the occurrence and distribution of oil spill in the incident site, and from the site to lagoon (Stars locate the incident site).

The developed RGB composites using the band ratios and MSI bands acquired before, during, and after the oil spill event between July 05 and September 15, 2020 are given in Fig. 2, Fig. 3. The (5 + 6)/7, (3 + 4)/2, and (11+12)/8 ratio images of July 17, 2020 acquired before the incident show the absence of Wakashio ship and oil spill in the incident site (star marked, Fig. 2(a)). But, the images acquired on August 01 and 06, 2020 (Fig. 2(b) and (c)), during the oil spill, show the occurrence of suspended sediments and oil spills in and around the ship. The suspended sediments that occurred during the aground of Wakashio exhibit bright yellow on the image dated August 01, 2020. The oil spills exhibit light blue (very thick oil spill) to bright orange (thick oil spill) to light orange (thin oil spill) with fine texture, and the spills are distinguishable over the image acquired on August 06, 2020. The distribution of spilled oil over the lagoon shows shades of light orange and the spread can be well interpreted. The image acquired on September 05, 2020, after the incident (the forward hull section of Wakashio was submerged, and no longer visible from the surface since August 24, 2020) [29] shows the spread of thin oil parallel to the coral reef ridge and the transport of oil away from the incident site (Fig. 2(d)). Similarly, the ratios 3/2, (3 + 4)/2, (6 + 7)/5 image of September 06, 2020 (Fig. 3(c)) show the oil spills in and around the ship and exhibits dark gray (very thick oil spill) to bright yellow (thick oil spill) to light orange (thin oil spill) with fine texture. The image of September 05, 2020 (Fig. 3(d)) exhibits the spilled oil that was transported from the incident site to near the coral reef ridge when compared to Fig. 2(d). Both the band ratios (5 + 6)/7, (3 + 4)/2, (11+12)/8 and 3/2, (3 + 4)/2, (6 + 7)/5 discriminated the oil spill of Wakashio very well.

### Decorrelation of Sentinel-2 bands to study oil spill thickness

**Fig. 4:**
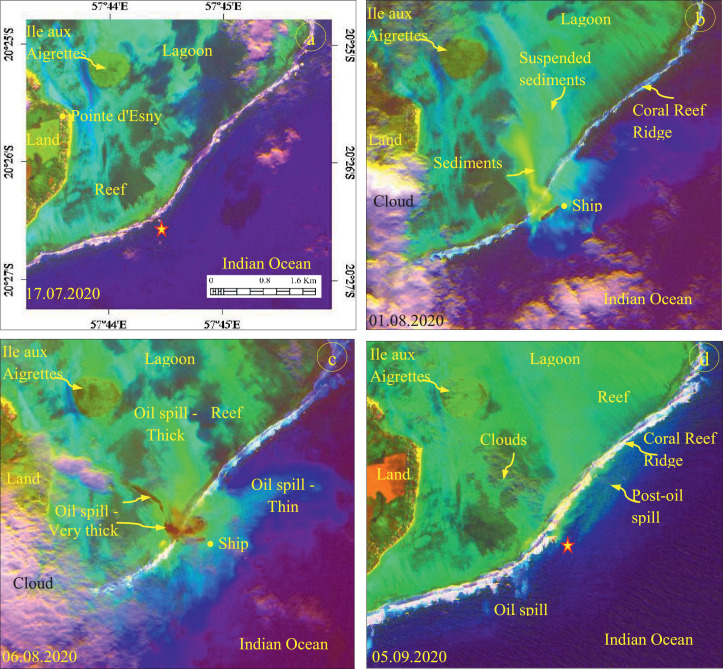
Decorrelated images of spectral bands 4, 3 and 2 of MSI showing the occurrence and spatial distribution of oil spill before, during and after the event and the oil spill from incident site to lagoon (Stars locate the incident site).

The images obtained by the decorrelation stretch method are given in Fig. 4. The image acquired on July 17, 2020, before the oil spill incident, shows the absence of Wakashio ship and oil spill in the incident site (star marked, Fig. 4(a)) similar to the results of the ratios images (Figs. 2(a) and 3(a)). But, the images acquired on August 01 and 06, 2020 (Fig. 4(b) and (c)), during the oil spill, show the occurrence of suspended sediments and oil spills distinctly. The suspended sediments appear in bright yellow with a fine texture. The occurrence and spatial distribution of the types of the oil spill can be interpreted from the different tones. The thin oil spill appears in light bluish-green, fine texture, and wide flow pattern distinctly in all the images. The thick oil spill exhibits a greenish-yellow, fine texture, and narrow flow pattern and the very thick oil spill exhibits brown tone, fine texture, and linear flow pattern in the images. The discriminations depend on the concentration of sediments in the water. The images show the spread of oil from the incident site towards the lagoon by crossing the coral reef ridge and confirm the spreading of oil in the offshore waters from the ship. The images clearly show the distribution of spilled oil and allow us to assess the thickness of the oil spills and their impacts in the lagoon and offshore of the study area. The image results can be compared with the band ratios of images (Figs. 2 and 3). The decorrelated images discriminated the thickness and spread of oil spills to the best [28]. However, the discrimination is also depending on the high spatial, spectral, and radiometric resolutions of the MSI of Sentinel-2.

## Conclusion

In this study, we developed the band ratios (5 + 6)/7, (3 + 4)/2, (11+12)/8 and 3/2, (3 + 4)/2, (6 + 7)/5 and discriminated the oil spill of Wakashio that occurred in the offshore region near the Pointe d'Esny, southeast coast of Mauritius using the MSI bands of Sentinel-2 acquired before, during and after the incident. The ratios were newly constructed by studying the image spectra of MSI bands and using the bands of 3 and 4, which are characteristics of the absorption of the oil spill. The developed band ratios discriminated the oil spills very well. Besides, the decorrelation stretch method applied to the MSI bands 4, 3, and 2 discriminated the very thick, thick, and thin type of oil spills and showed the spread of spill from the incident site to the coast. This study demonstrated the potential use of the developed band ratios and decorrelation stretch methods to discriminate and assess the thickness and impacts of the oil spill. The methods developed in this work are recommended to use for similar spectral bands of other sensors to map oil spills.

## Declaration of Competing Interest

The authors declare that they have no known competing financial interests or personal relationships that could have appeared to influence the work reported in this paper.

## Appendix Supplementary materials

Image, application 1
