# Supplementary material for: Sentinel-2 image transformation methods for mapping oil spill – A case study with Wakashio oil spill in the Indian Ocean, off Mauritius
Source: MethodsX. 2021 Mar 27;8:101327. doi: 10.1016/j.mex.2021.101327 (PMC8374399; doi:10.1016/j.mex.2021.101327)
Supplement: Supplementary file 1 [file mmc1.docx]

**Appendix-A**

**Table 1**

Sentinel-2 data used to demonstrate the methods.

| Sensor | Date of Acquisition | Image ID | Cloud cover % |
| --- | --- | --- | --- |
|  |  |  |  |
| Sentinel-2  MSI | 10092020 | S2A_MSIL1C_20200910T062451_N0209_R091_T40KEC_20200910T073822 | 20.6178 |
|  | 05092020 | S2B_MSIL1C_20200905T062449_N0209_R091_T40KEC_20200905T075219 | 12.4964 |
|  | 16082020 | S2B_MSIL1C_20200816T062449_N0209_R091_T40KEC_20200816T074432 | 4.4146 |
|  | 11082020 | S2A_MSIL1C_20200811T062451_N0209_R091_T40KEC_20200811T094614 | 1.8165 |
|  | 06082020 | S2B_MSIL1C_20200806T062449_N0209_R091_T40KEC_20200806T084627 | 21.3481 |
|  | 01082020 | S2A_MSIL1C_20200801T062451_N0209_R091_T40KEC_20200801T085936 | 15.5289 |
|  | 17072020 | S2B_MSIL1C_20200717T062449_N0209_R091_T40KEC_20200717T093058 | 4.7543 |
|  | 19032020 | S2B_MSIL1C_20200319T062449_N0209_R091_T40KEC_20200319T093014 | 0.0312 |

**Fig. 1.** (a) Relative reflectance of oil and seawater (after Fingas and Brown, 2017), (b) the MSI image (R:4; G:3; B:2) showing the locations of very thick, thick and thin oil spill, (c) the image spectra of lagoon (Shallow water: Cyan; Deep water: Blue) and offshore (Ocean water: Dark blue) and (d) the image spectra of oil spill (Very thick oil: Blue; Thick oil: Magenta; Thin oil: Green).


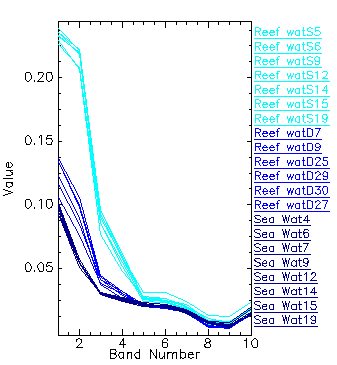

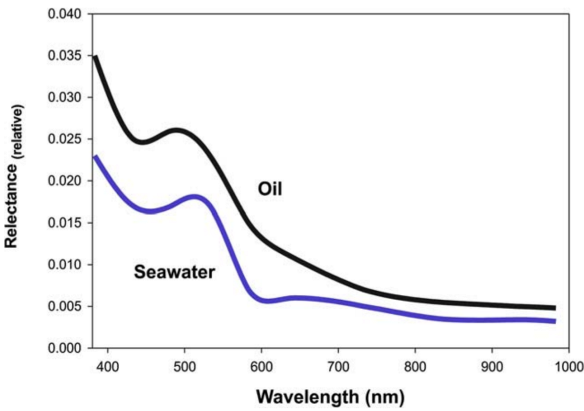

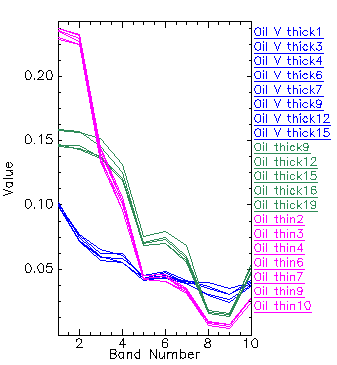

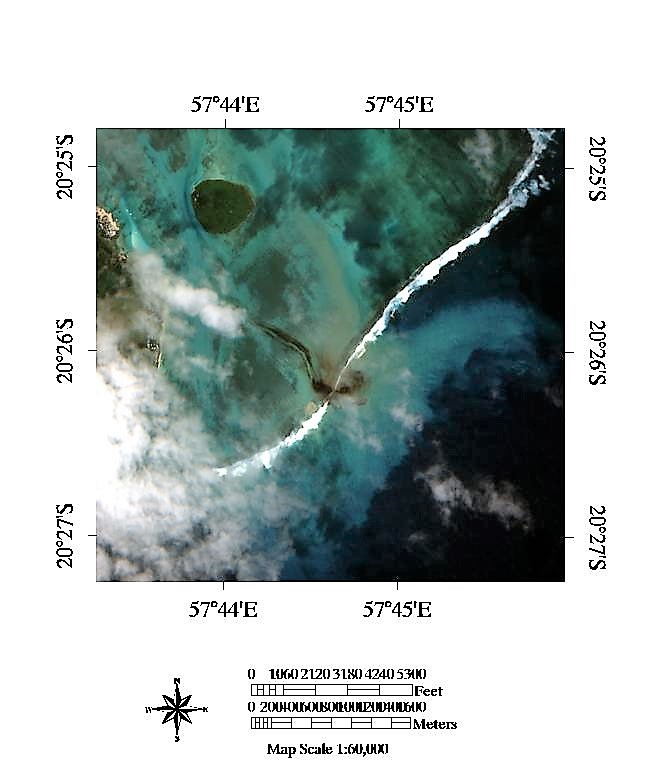


•Ship

06.08.2020

Oil spill –

Thin

Indian Ocean

•

Ile aux Aigrettes

Lagoon

Oil spill - thick

Oil spill -

Very thick

Coral Reef ridge

Offshore

**Appendix-1**
